# Mitigating thermal runaway in EV batteries using hybrid energy storage and phase change materials

**DOI:** 10.1039/d5ra02870a

**Published:** 2025-07-16

**Authors:** Mohammad Talha, Rupesh Palange, Saleem Anwar Khan, Cataldo DeBlasio

**Affiliations:** a Laboratory of Energy Technology, Åbo Akademi University 65100 Vaasa Finland rupesh.palange@abo.fi; b Department of Mechanical Engineering, Aligarh Muslim University 202002 Aligarh India

## Abstract

Electric vehicles (EVs) are increasingly recognized as a sustainable solution for modern transportation; however, effective thermal management of their battery systems is essential to ensure safety, reliability, and optimal performance. This review examines advanced strategies for preventing thermal runaway in EV battery systems, with a focus on innovative thermal management techniques. It introduces various battery chemistries suitable for different applications and highlights key thermal control methods, including the use of phase change materials (PCMs), heat sinks, and hybrid energy storage systems (HESS). Particular attention is given to HESS as a novel approach that integrates battery packs with metal-hydride tanks for improved thermal regulation. Furthermore, the paper presents a comprehensive analysis of different battery thermal management system (BTMS) configurations, emphasizing their critical role in enhancing both the safety and operational efficiency of electric vehicles.

## Abstract 2

Working diagram of HESS with battery pack and metal hydride tank.

## Graphical abstract

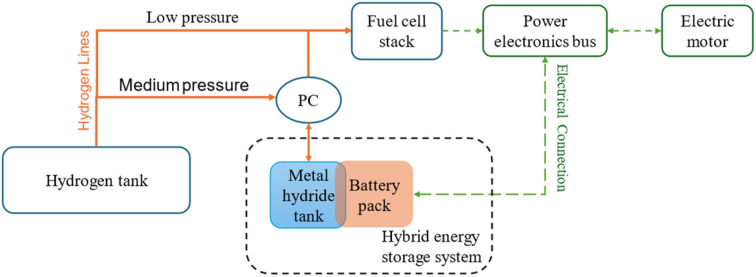

## 1. Introduction

Greenhouse gas emissions are reaching alarming levels due to the rapid expansion of industries and transportation worldwide. One of the reasons is that a lot of the energy storage options available today depends on fossil fuels.^1^ Approximately 29% of global greenhouse gas emissions are attributed to the transportation sector, highlighting its significant role in climate change.^2^ With increase in consumer demand and advancement in production technology there has been steady rise in the number of vehicles on road. There were approximately 50 million vehicles worldwide in 1950, equating to about two vehicles for every 100 people. By 1994, this number had increased to around 600 million, or roughly 10 vehicles per 100 people. If current trends continue, the global number of automobiles could exceed 3 billion by 2050 equivalent to more than 20 vehicles for every 100 people.^3^ These vehicles contribute significantly to urban pollution, emitting increasing amounts of greenhouse gases along with carbon dioxide and substantial quantities of petroleum-based pollutants. This is contributing to climate change and harming the environment. So, this necessitates a shift towards electric vehicles (EVs) and raising public awareness about their benefits. Because battery-powered EVs have a positive impact on energy consumption and atmospheric air quality, there has been a global focus on developing better rechargeable batteries for these vehicles.^4^ The popularity of electric vehicles (EVs) is rapidly increasing, with record-breaking sales in 2022. With over 10 million EVs were sold that year, accounting for 14% of all new car sales up from 9% in 2021. As a result, more than 26 million electric cars are now on the road globally, marking a 60% rise compared to the previous year. This growth positions the transportation sector as a key player in addressing climate change and advancing the United Nations Sustainable Development Goals (SDGs). Introduced in 2015, the 17 SDGs represent a global vision for achieving a high quality of life, environmental sustainability, and long-term peace for all.^5^ SuM4All's Global Roadmap of Action (GRA) for sustainable mobility outlines the need to reduce greenhouse gas emissions from the transport sector from 8 billion tons of CO_2_ to 2–4 billion tons by 2050, with the long-term objective of achieving net-zero emissions in the following decades.^6^ Another significant step toward achieving sustainable transportation is the ‘60 km rule’ introduced by the European Union. This regulation mandates that member states install fast-charging stations with a minimum output of 150 kW every 60 kilometres along the trans-European transport network. Additionally, it requires a minimum charging capacity of 1.3 kW per registered battery electric vehicle (BEV). For heavy-duty vehicles, the 60 km rule also applies; however, the minimum power output for charging stations must be at least 350 kW.^7^ These charging stations are typically charged using solid oxide fuel cells (SOFC). SOFC have also popularly found applications in propulsion of hybrid electric vehicles.^8^ The energy efficiency of these systems can be optimized by integrating them with syngas and different multifuel systems.^9–12^ However, with rise in the number of electric vehicles issues have been raised with vehicle safety and battery longevity. Batteries must perform reliably across all climates to ensure the widespread adoption of EVs amidst the rapidly changing global weather patterns. The primary challenge in battery system design lies in the thermal behaviour of the battery pack, with lithium-ion batteries being the preferred choice due to their high energy density and long cycle life.^13,14^ Nonetheless, these batteries present significant thermal management challenges, including capacity and power degradation, the risk of thermal runaway, electrical imbalances among cells, and suboptimal performance at low temperatures. Different studies have emphasized the critical role of temperature and thermal management in mitigating these issues, particularly with regards to capacity fade, thermal runaway, and intra-pack electrical imbalances. Thermal runaway in electric vehicles is typically triggered by a rapid and uncontrolled rise in battery temperature, initiating an exothermic reaction that further accelerates the temperature increase. This self-reinforcing process can result in irreversible damage to the battery system. For large prismatic lithium-ion cells, thermal runaway may be initiated at approximately 870 °C in a 25 Ah battery, potentially resulting in an explosive event.^15^ Such incidents pose severe safety risks and may be fatal. According to the Federal Aviation Administration, 64 aviation-related incidents involving lithium batteries transported as cargo or baggage were reported in 2022. Furthermore, between March 3, 2006, and April 24, 2024, 100 aviation-related incidents were recorded involving lithium batteries associated with e-cigarettes.^16^Table 1 lists a few battery explosion incidents that have occurred in the past years.

**Table 1:** Incidence of battery explosions

Date	Location	Incident	Cause	References
August 1, 2024	Incheon, South Korea	Fire in Mercedes-Benz electric car	Explosion in battery	17
April 4, 2024	Charlotte to Las Vegas flight	In-flight emergency due to smoke	Passenger's e-cigarette battery	18
January 10, 2018	Spain	iPhone explosion	Overcharging	18
May 17, 2018	China	Electric bus fire	Overdriving, battery line aging, short circuit	18
June 24, 2024	South Korea	Factory fire with 22 fatalities	Lithium battery explosions	19
September 19, 2023	Delhi, India	E-scooter battery explosion	Charging of battery	20
September 30, 2023	Bengaluru, India	Electric car fire	Battery heating	21

A significant number of studies have also reported the causes of battery abuse and proposed solutions to enhance their efficiency and effectiveness as energy storage solutions. One key focus is on developing strategies to mitigate thermal runaway. Xuning Feng *et al.*^22^ have provided a comprehensive overview of various mitigation strategies for thermal runaway in lithium-ion batteries, addressing interventions at the material, cell, and system levels. At the cell level, their proposed time-sequence map elucidates the temporal relationship between the onset of thermal runaway and subsequent fire events. At the system level, the map further illustrates the expected propagation behaviour of thermal runaway and highlights potential pathways for unintended fire spread. Spotnitz and Franklin^23^ conducted comprehensive investigations into the abuse tolerance of lithium ion batteries by developing a series of standardized test to examine the behaviour of battery under extreme conditions. These tests include short circuit test, oven test, overcharge test, and nail and crush test. It was found that the rapid and localized heating of the battery was induced during the short circuit and nail and crush tests. This leads to elevated temperatures that initiate the major chemical reactions within the cell. Ensuring effective thermal management is critical for the safety, reliability, and high performance of EV battery systems. Operating batteries within an optimal temperature range not only enhances energy density and power output but also minimizes degradation, thereby significantly extending battery lifespan. Despite growing advancements in battery technologies, the risk of thermal runaway remains a major concern, necessitating more sophisticated and adaptive thermal control solutions. This review provides a comprehensive and timely examination of cutting-edge strategies for mitigating thermal runaway in EVs, with a particular emphasis on innovative thermal management techniques that implement the use of hybrid energy storage systems.

The proposed hybrid energy storage system (HESS) integrates lithium-ion battery packs with metal hydride tanks and phase change materials (PCMs), presenting an innovative approach to thermal management in EVs. Metal hydride tanks, traditionally employed for hydrogen storage, offer a unique passive thermal regulation mechanism through their endothermic hydrogen release and exothermic absorption processes.^24^ By coupling this system with PCMs, the HESS can enhance heat absorption and dissipation, overcoming key limitations of standalone PCM-based battery thermal management systems, such as low thermal conductivity and susceptibility to vibrational effects.^25^ Vibration is unavoidable in EVs and hybrid electric vehicles (HEVs), where it's intended to implement the proposed thermal management systems. Khan *et al.*^25^ discussed the impact of vibrations on PCM. Unlike conventional hybrid BTMS that rely on energy-intensive active cooling methods (*e.g.*, air, liquid, or thermoelectric cooling), this hybrid system uses passive thermal management, improving energy efficiency and system simplicity.

With advancements in fast-charging and high-rate discharging technologies, traditional PCM-based BTMS face challenges due to excessive heat accumulation and localized temperature spikes in battery packs, primarily caused by PCMs' inherently low thermal conductivity (*e.g.*, 0.2 W m^−1^ K^−1^ for paraffin-based PCMs).^26^ To address this, researchers have focused on enhancing PCM thermal conductivity through additives, broadly categorized into carbon-based materials (*e.g.*, expanded graphite) and metal-based materials (*e.g.*, copper grids).^27^ Preliminary studies indicate that incorporating composite PCMs with 12% expanded graphite into the HESS can reduce temperature gradients by up to 30% compared to conventional PCM-based systems.^27^ This enhancement ensures uniform temperature distribution, which is critical for maintaining battery performance and safety during high-rate operations. The passive nature of the HESS reduces reliance on complex active cooling systems, lowering costs and improving scalability. This approach is particularly effective for managing the thermal demands of lithium-ion batteries under dynamic operating conditions, such as fast charging, making it a promising solution for next-generation EVs. The proposed system not only enhances the efficiency and safety of EV battery thermal management but also contributes to the sustainability of electric mobility by reducing energy consumption in thermal regulation. Subsequent sections of this article will further explore the performance, design considerations, and practical implementation of this innovative system.

## 2. Classification of battery technologies

Batteries are energy storage devices that utilize electrochemical oxidation and reduction reactions to convert the chemical energy within their active materials into electrical energy. They are a crucial part of today's energy systems and are the fastest-growing technology in the energy market. Lithium-ion batteries are still the most widely used in electric vehicles (EVs) and other energy storage systems. However, battery designs are changing to make better use of available materials and reduce costs. One example is lithium iron phosphate (LFP) batteries, which made up 40% of EV sales and 80% of new battery storage installations in 2023.^28^

Battery technologies can be classified based on the following characteristics:

(1) Primary batteries: these are non-rechargeable batteries and are disposed of after use. These are often “dry cells” with a paste-like electrolyte.

(2) Secondary batteries: rechargeable batteries that allow the electrochemical reaction to be reversed by applying a reverse current, enabling multiple charge–discharge cycles.

(3) Power density: it measures the power output per unit weight (W kg^−1^), determining how quickly energy can be delivered.

(4) Energy density: it refers to the amount of energy stored per unit weight (Wh kg^−1^), calculated by integrating the current and voltage over a full discharge cycle.

(5) Energy efficiency: the ratio of discharged energy to charged energy, with losses converted into heat, which must be managed to prevent overheating.

(6) Cycle life: the number of charge–discharge cycles a battery can undergo before its performance degrades, influenced by factors like cycle depth, current rate, and state of charge (SOC).

(7) Cost: includes the initial purchase price, along with costs for charging and maintenance over the battery's lifespan.

### 2.1 Li-ion battery

**Fig. 1:**
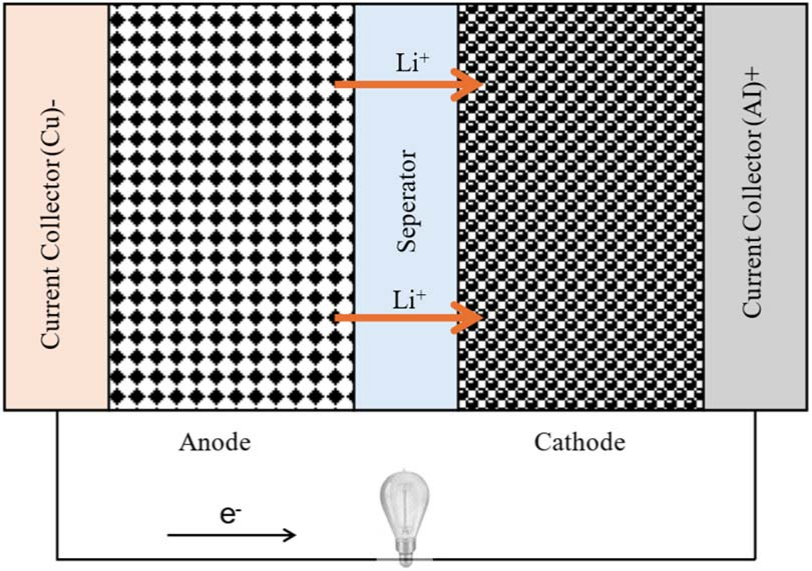
Li-ion battery.

Lithium-ion batteries are the most common commercial rechargeable batteries used in portable devices and electrified transportation. According to the specifications of different battery technologies at the cell level discussed by Budde-Meiwes *et al.*,^29^ it is evident that, in comparison to other battery technologies, Li-ion batteries have a high energy density (between 50 Wh kg^−1^ and almost 200 Wh kg^−1^) and a high specific power. Li-ion batteries have a cell voltage that ranges from 3.3 to 3.7 V.^30^ For HEVs, the cost is relatively high, ranging from 400 to 800 euros per kWh.^29^Fig. 1 illustrates a schematic diagram of the electrochemical components inside a Li-ion battery. During the discharging process, the positively charged lithium ions move from the negative anode to the positive cathode by traveling through the electrolyte until they are deposited at the cathode. Simultaneously the electrons flow through the external circuit from anode to cathode to complete the electrical circuit and facilitating the energy transfer.

The energy conservation equation, which determines temperature variation in a Li-ion battery,^31^ is expressed as:1
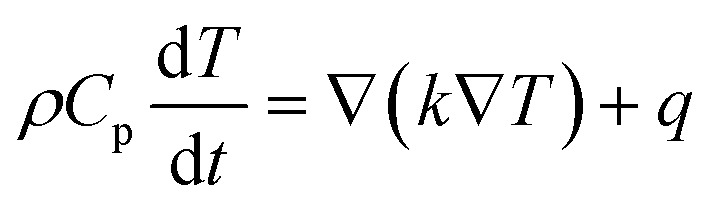
In this equation, the left-hand term represents the heat stored within the battery due to its specific heat capacity (*C*_p_) and density (*ρ*). The term ∇(*k*∇*T*) accounts for heat conduction within the battery, influenced by its thermal conductivity (*k*). The heat generation rate (*q*) includes contributions from irreversible losses such as internal resistance, electrochemical reactions, and reversible heat effects related to entropy changes.

**Fig. 2:**
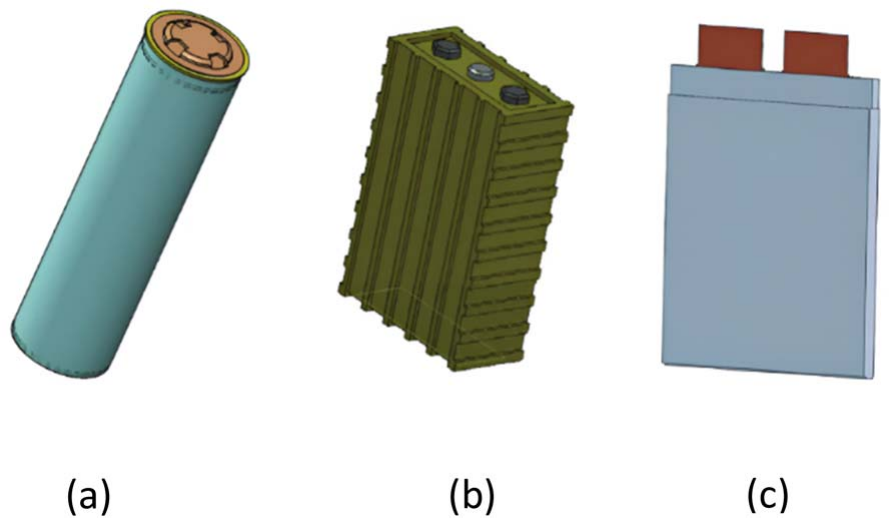
Cell designs: (a) cylindrical, (b) prismatic, (c) pouch.

One of the most crucial battery states to monitor is the state of charge (SoC) to maximize performance and prolong battery life. The SoC of a battery refers to the proportion of the battery capacity that remains after it is fully charged. It is defined as the rate of available capacity to its maximum capacity. A summary of the techniques for estimating SoC for lithium-ion batteries was provided by Rivera-Barrera *et al.*^32^ Further, to supply high power from the batteries, an electrolyte with high lithium-ion conductivity and low resistance across the electrolyte–electrode interface is necessary.^33^ Li-ion batteries have many applications because of their advantageous qualities. It is compatible with mobile phones, EVs, laptops, and other portable electronics. These batteries are made with a variety of cell configurations, including cylinder, prismatic, and pouch cells (Fig. 2). Cylindrical battery cells are a common choice for EV battery packs due to their high energy density, which is crucial for achieving long driving ranges. By combining these cells in both series and parallel configurations, manufacturers can optimize the pack's voltage and capacity to meet specific vehicle requirements. Tesla's Model S is a prime example, utilizing a series-parallel arrangement of 18 650 lithium-ion batteries.^34^ This configuration allows the pack to reach a high voltage, providing sufficient power for the vehicle's performance, and a substantial capacity, ensuring a long driving range. Today's EVs typically operate at voltages between 400 V and 800 V,^35^ and battery pack capacities range from 40 kWh to over 100 kWh, depending on the desired range and other factors. A study by Geo *et al.*^36^ on retired Nissan Leaf battery packs found that, despite a significant capacity loss (60–67%), these packs can still have a useful lifespan of 12–20 years in second-life applications. This suggests that there is considerable value to be extracted from retired EV batteries, with potential economic benefits.

### 2.2 Sodium-ion battery (Na-ion)

Na-ion batteries are emerging as a promising alternative to Li-ion batteries, offering similar performance while potentially reducing costs and environmental impact. These batteries work similar to Li-ion batteries with exchange of sodium ions between the positive and negative electrodes. Because it depends on more readily available materials for manufacturing, Na-ion technology is a very attractive choice. Metal oxides, phosphates and organic material are utilized to make the cathode materials for Na-ion batteries, while recent technological developments have promoted use of carbonaceous materials, transition metal sulphides, intermetallic and organic compounds as anode materials (Table 2).^37,38^

**Table 2:** Specifications of Na-ion battery

Voltage	Energy density	Cycle life	Operating temperature	Safety	Cost (€ per kWh)
3.4 V per cell (ref. 30)	100–160 Wh kg^−1^ (ref. 39)	Over 5000 cycles at 87.5% capacity retention^37^	−20 to +60 °C (ref. 39)	Lower reactivity ensures safety compared to Li on battery^37^	223 (ref. 40)

### 2.3 Lead–acid battery

Lead–acid batteries are a mature technology used in cars, trucks, and stationary energy storage systems. When compared to Li-ion and Na-ion batteries, they are less expensive but have a lower energy density and a shorter cycle life. Early efforts to develop lead acid batteries started in 1880s. In 1881, Gustave Trouve utilized a lead–acid battery for the first time to power a three-wheeled electric automobile, which reached a speed of 12 km h^−1^. The first submarines to be propelled by lead acid batteries were developed in 1886.^41^ Lead–acid sealed pack batteries are susceptible to the thermal runaway effect since they include a liquid electrolyte, but the likelihood of this happening is lower than with Li-ion batteries (Table 3).

**Table 3:** Specifications of lead–acid battery

Voltage	Energy density	Specific energy	Power density	Cycle life	Self-discharge	Maintenance	Cost (€ per kWh)
Typically, 2 V per cell (ref. 30)	30–40 Wh kg^−1^,^30^ lower than Li-ion and Na-ion batteries	20–35 Wh kg^−1^	180 W kg^−1^	Limited compared to Li-ion and Na-ion batteries	Moderate	Require regular maintenance, such as checking electrolyte levels	25–40

### 2.4 Nickel–metal hydride (NiMH) battery

NiMH batteries were once popular in hybrid cars and portable electronics but have been largely replaced by Li-ion batteries. Higher energy density, high power, longer lifespan, abuse tolerance, a wide working temperature range, quick charging capability, and completely sealed maintenance-free operation are among the benefits they offer. These batteries store hydrogen in a solid form, and the amount of hydrogen they can hold determines how much energy they can store.^42^ The manufacturer recommends storing the battery at a low charge level, like C/30 or C/40, to reduce internal stress and decrease gas generation over time.^43^ Charging at these lower rates is less aggressive on the battery, which helps maintain its longevity and safety during long-term storage (Table 4).

**Table 4:** Specification of NiMH battery

Voltage	Energy density	Cycle life	Self-discharge	Negative electrode	Positive electrode	Electrolyte	Cost (€ per kWh)
Typically 1.2 V per cell (ref. 42)	170 Wh L^−1^ (ref. 44)	Shorter than Li-ion batteries	Moderate	MH^44^	NiOOH^44^	KOH^44^	275–550 (ref. 29)

### 2.5 Nickel–cadmium (NiCd) battery

NiCd batteries used to be popular but have been replaced by NiMH and Li-ion due to cadmium being harmful. Unlike other batteries, NiCd plates don't corrode or deteriorate, so they can be made thinner. This makes them more expensive per ampere hour but also more efficient in high-rate discharges, making them cost-effective overall (Table 5).^45^

**Table 5:** Specification of NiCd Battery

Voltage	Energy density	Cycle life	Self-discharge	Negative electrode	Positive electrode	Electrolyte	Specific energy	Cost (€ per kWh)
1.2 V per cell (ref. 30)	60–80 Wh kg^−1^ (ref. 30)	Over 6000 cycles at 80% depth of discharge^45^	Moderate	Cadmium (Cd)	NiOOH	KOH	50 Wh kg^−1^	200–500 (ref. 29)

These batteries can be used for various purposes, including EVs. However, this study focuses on lithium-ion batteries due to their unique qualities and benefits. To ensure optimal performance and safety, these batteries require special thermal management systems to control their internal temperature according to the specific application. In the following sections, existing and potential thermal management techniques will be discussed.

## 3. Battery thermal management techniques

Effective battery thermal management is crucial for maintaining optimal battery performance and the sustainability of electric vehicles by reducing temperature disparities between cells. Traditional cooling methods include air cooling, liquid cooling, and use of phase change materials. Thermal management is generally not a significant concern for batteries at low discharge rates, as indicated by Chen *et al.*^46^ Their study on lithium/polymer-electrolyte batteries demonstrates that operating at low current densities, combined with a significant temperature gradient between the battery and its environment, can effectively mitigate the risk of overheating. There has also been growing interest in using HESS for thermal management purposes. HESS combines a battery pack with a metal hydride-based hydrogen storage system, using the endothermic desorption process of hydrogen in metal hydrides to manage battery temperature efficiently.^47^ This integration presents a promising approach to thermal management in battery systems. Passive and active thermal management techniques are the two categories of battery thermal management strategies. These are covered in more detail in further sections.

### 3.1 Passive thermal management techniques

Passive thermal management systems do not require mechanical moving parts or active fluid circulation, resulting in zero power consumption and low mass and cost. These systems are highly reliable due to their simple design, ease of implementation, and straightforward testing processes. However, a key drawback of passive thermal management techniques is their generally low heat transport capability compared to active thermal management systems, except heat pipes, which can offer relatively high thermal conductivity. Due to the advantages of passive thermal management, significant research is being conducted in this area.

#### 3.1.1 Thermal management by phase change materials

PCMs are found to have widespread applications for the thermal management of batteries due to their high latent energy storage capacity. To enhance their thermal properties, such as thermal conductivity and specific heat, PCMs are often combined with nanomaterials. This integration significantly boosts the energy absorption capacity of the materials, leading to notable temperature increases during the phase change process. The thermal conductivity of PCMs can be improved by incorporating highly conductive nanoparticles, expanded graphite, metal foams, or through PCM encapsulation. For example, in a study by Paul *et al.*,^48^ a nano-enhanced PCM was developed by dispersing hybrid graphene-silver nanofillers into paraffin at varying concentrations (0.1%, 0.3%, and 0.5%). The results showed a maximum increase of 6.7% in latent heat and a 5% improvement in heat storage efficiency for the nanocomposite with 0.3 wt% of additives. Table 6 outlines various enhancement techniques for improving the thermal conductivity of PCMs, as explored by researchers.

**Table 6:** Techniques for improving the thermal conductivity of PCMs

Enhancement methods	Specific technique
Addition of nanoparticles	Carbon nanotubes^49^
	Graphene nanoparticles^50^
	Silver nanoparticles^51^
Impregnation methods^52^	Melt blending (paraffin with expanded graphite)^53^
	Melt blending (PCM mixed with Al_2_O_3_)^54^
	Vacuum impregnation^55^
Metal foam^56^	Nickel foam^57^
	Copper foam^58^
	Graphite foam^59^
	Aluminium foam^56^
Encapsulation	Porous matrix absorption PCM^60^
	Microencapsulated PCM^61^
Other methods	Hexagonal boron nitride^62^
	Spongy graphene^63^

In a study on passive thermal management, Mills and Al-Hallaj^64^ developed a PCM where paraffin wax is used in combination with expanded graphite matrix. It was revealed that because the battery temperature exceeded 55 °C during a P/1.25 discharge, there was a need to increase the volume of the PCM with expanded graphite. Verma *et al.*^65^ investigated the use of capric acid as a PCM. They applied PCM layers of varying thicknesses of 3 mm, 7 mm, 9 mm, and 12 mm around the periphery of the battery pack. Their findings indicated that a 3 mm layer of capric acid successfully reduced the temperature to 305 K, which was more effective compared to the 9 mm layer of paraffin wax. Various researchers have conducted similar studies on the impact of PCM thickness. Talha and Khan,^66^ for instance, explored the effect of varying PCM thickness using paraffin wax combined with nano graphite. The investigations concluded that the temperature distribution was more stable for the 7 mm PCM layer thickness. *N*-octadecane was the PCM utilized by Javani *et al.*^67^ for different thickness ranging from 3 mm to 12 mm around the Li-ion battery to estimate the distribution of temperature fluctuations. The maximum temperature is lowered by 3.04 K by using PCM which is 12 mm thick. The 3 mm, 6 mm, and 9 mm layers have equivalent values of 2.77 K, 2.89 K, and 2.98 K, respectively. It was interesting to note that the point of peak temperature shifts closer to cell's interior with increase of PCM layers around the cell. Without the PCM, this point is closer to the cell's bottom side. For Li-ion battery applications, the ideal PCM should have a narrow melting temperature range, a high latent heat capacity, and a melting point ranging between 30 °C and 60 °C. Hallaj and Selman^68^ developed a comparative study between a traditional battery cooling methods and use of a paraffin wax as PCM. The space between the cells in the battery module were filled with paraffin wax. It was observed that the temperature increase for the PCM was only 11 K, whereas the temperature at the core of the cells reached nearly 53 K by the end of discharge. A PCM-assisted heat pipe was proposed by Behi *et al.*^69^ to improve cooling and reduce internal heat loss in electronic devices. Their research showed that the module could greatly lower the risk of thermal damage to electronic components by providing 86.7% of the cooling and preventing 11.7% of heat loss through extra heat absorption. Table 7 presents the materials used for battery thermal management, their specifications, and details of the battery packs utilized by various researchers in their studies. When heat is transferred through a phase change material (PCM), the solid and liquid phases coexist during the phase change process. Javani *et al.*^67^ studied this by implementing a mathematical model based on enthalpy-porosity calculations. In this approach, instead of explicitly tracking the melting interface in the mushy zone, the liquid fraction represents the portion of a cell in the liquid state.^70^ A linear source term, along with a linear variation in the liquid fraction, is assumed across the melting/solidification interface. This modelling assumption leads to an effective porosity of 0.5 at the liquid–solid boundary.

**Table 7:** Materials and their specifications

Reference	Cooling materials	Material specifications			Battery pack details
Mills and Al-Hallaj^64^	Paraffin wax (Rubitherm RT-42) with expanded graphite matrix	Thermal conductivity: 16.6 W m^−1^ K^−1^			18 650 Li-ion battery. Pack composed of six 2.2 Ah Li-ion battery cells
		Latent heat: 127 J g^−1^			
		Specific heat: 1.98 J g^−1^ K^−1^			
		Density of composite: 7.89 × 10^5^ g m^−3^			
		Density of graphite: 2.10 × 10^5^ g m^−3^			
Verma *et al.*^65^	Capric acid	Heat capacity: 475.59 J kg^−1^ K^−1^			Li-ion battery, thermal conductivity: 25 W m^−1^ K^−1^, density: 1881 kg m^−3^, specific heat: 1097 J kg^−1^ K^−1^
		Latent heat: 152.7 kJ kg^−1^ K^−1^			
		Density: 878 kg m^−3^			
		Thermal conductivity: 0.153 W m^−1^ K^−1^			
		Solidus temperature: 302 K			
		Liquidus temperature: 305 K			
		Boiling temperature: 542 K			
Javani *et al.*^67^	*N*-octadecane	Solidus temperature: 301.15 K			Thermal conductivity: 25 W m^−1^ K^−1^, specific heat: 1027 J kg^−1^ K^−1^ , heat generation rate: 63.970 kW m^−3^, 2C (C-rate)
		Liquidus temperature: 303.15 K			
		Specific heat (J kg^−1^ K^−1^)	2150 (solid phase)		
			225 000 (mushy zone)		
			2180 (liquidus phase)		
		Thermal conductivity (W m^−1^ K^−1^)	0.358 (solid phase)		
			0.255 (mushy zone)		
			0.152 (liquidus phase)		
		Density (kg m^−3^)	814 (solid phase)		
			769 (mushy zone)		
			724 (liquidus phase)		
Talha and Khan^66^	Paraffin wax with nano graphite	Heat capacity: 475.59 J kg^−1^ K^−1^			Li-ion battery with 2C (C-rate), heat generation rate: 63 970 W m^−3^
		Density: 840 kg m^−3^			
		Thermal conductivity: 0.5685 W m^−1^ K^−1^			
		Solidus temperature: 297 K			
		Liquidus temperature: 302 K			
Behi *et al.*^71^	Paraffin wax	Phase change temperature: 30 °C			LTO battery with 30 cells, prismatic in shape, nominal voltage: 2.3 V, capacity: 23 Ah, specific energy: 96 Wh kg^−1^, energy density: 202 Wh L^−1^
		Melting point: 25–32 °C			
		Heat storage capacity: 220 kJ kg^−1^			
		Specific heat capacity: 2.5 kJ kg^−1^ K^−1^			
		Density at 15 °C: 0.8 kg L^−1^			
		Density at 80 °C: 0.85 kg L^−1^			
		Thermal conductivity-solid: 0.25 W m^−1^ K^−1^			
		Thermal conductivity-liquid: 0.4 W m^−1^ K^−1^			
Khaboshan *et al.*^72^	*n*-Eicosane	Density, kg m^−3^	910 (solid phase)		18 650 Li-ion battery (Panasonic NCR 18650 PF, 2.4 Ah), heat generation rate: 94 023.8 W m^−3^, 3C discharge rate
			769 (liquid phase)		
		Thermal conductivity, W m^−1^ K^−1^	0.423 (solid phase)		
			0.146 (liquid phase)		
		Specific heat capacity, J kg^−1^ K^−1^	1926 (solid phase)		
			2400 (liquid phase)		
		Melting point: 309.55 K			
		Latent heat of fusion, 248 kJ kg^−1^			
Bais *et al.*^73^	Paraffin wax (RT-42)	Thermal conductivity: 0.2 W m^−1^ K^−1^			18 650 Li-ion cell, 3.7 V, 1.5 Ah, 3C discharge rate
		Solidus temperature: 311 K			
		Liquidus temperature: 316 K			
		Density: 880 kg m^−3^			
		Specific heat: 2000 J kg^−1^ K^−1^			
		Latent heat: 165 000 J kg^−1^			
Zhang *et al.*^74^	Sulfur-free expanded graphite/paraffin (EG/PA-5.0)	Thermal conductivity: 2.56 W m^−1^ K^−1^			Lithium iron phosphate (LiFePO_4_) battery, 3.0–3.3 V per cell, 1C discharge rate
		Latent heat of phase transition: 253.08 J g^−1^			
Arshad *et al.*^75^	RT-35HC	Melting temperature: 308.15 K			Electronic devices such as integrated circuits, which generate high heat fluxes (25–40 kW m^−2^)
		Liquidus temperature: 309.5 K			
		Solidus temperature: 306.5 K			
		Latent heat of fusion: 240 000 J kg^−1^			
		Density: 825 kg m^−3^			
		Specific heat capacity: 2000 J kg^−1^ K^−1^			
		Thermal conductivity: 0.2 W m^−1^ K^−1^			
Tariq *et al.*^50^	RT-44HC and RT-64HC with nano-enhanced by graphene nanoparticles		**RT-44HC**	**RT-64HC**	Electronic devices with heat fluxes: 0.86 kW m^−2^, 1.44 kW m^−2^, 2.40 kW m^−2^
		Melting temperature	314–317 K	336–338 K	
		Heat storage capacity	2.0 kJ kg^−1^	2.0 kJ kg^−1^	
		Density	800 kg m^−3^	880 kg m^−3^	
		Thermal conductivity	0.2 W m^−1^ K^−1^	0.2 W m^−1^ K^−1^	

The liquid fraction is updated in each iteration based on the enthalpy balance equation:2
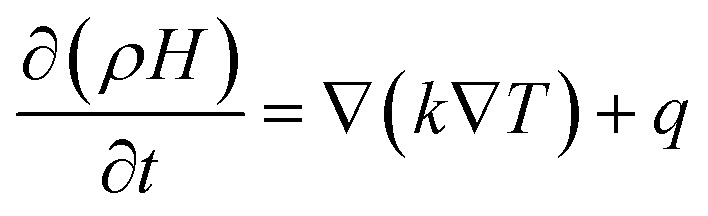
Here, the enthalpy of the PCM (*H*) is expressed as:

3 *H* = *h* + Δ*H*

where *H* is the total heat content, *h* is the sensible heat, and Δ*H* is the material's latent heat. In the calculation domain, the heat generation rate (*q*) is constant within the cell zone and zero in the PCM region. This method simplifies phase change modeling by integrating enthalpy changes and their corresponding impact on temperature distribution.

#### 3.1.2 Thermal management using heat sinks

**Fig. 3:**
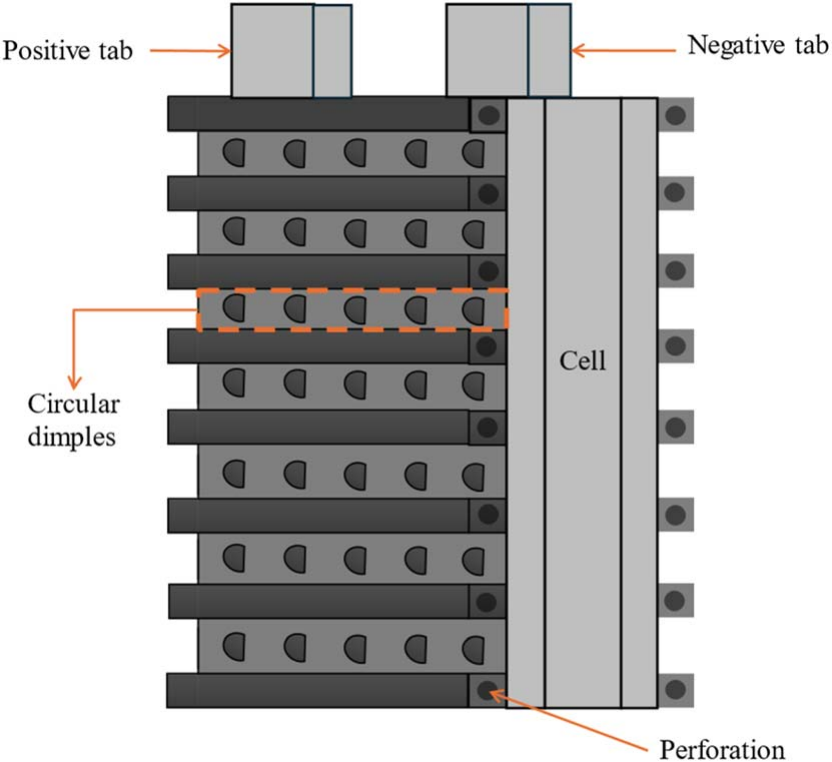
Battery with the heat sink.

The heat sinks employed in Battery Thermal Management Systems (BTMS), are typically consist of high-conductivity materials such as aluminium or copper, which are highly effective at absorbing and dissipating heat. Behi *et al.*^76^ demonstrated the use of aluminium heat sink for thermal management of a LTO prismatic battery cell at an 8C discharge rate. They compared natural convection (NC), NC with a heat sink, and forced convection. The maximum heat sink temperature reached 51.5 °C with NC and 31.1 °C with forced convection, reducing the cell's temperature by 10% and 44.4%, respectively. A filler material with thermal conductivity of 8 W m^−1^ K^−1^ is used to reduce the thermal contact resistance between heat sinks and cells. These fillers enhance thermal contact by filling microscopic air gaps between the battery cells and the heat sink, thereby improving heat transfer. Within the heat sink, heat is transferred primarily through conduction, convection, and to a lesser extent, radiation. The heat generated by the battery cells is conducted to the heat sink through direct contact, where it is then distributed across the heat sink. This heat is subsequently released into the surrounding environment, often with the assistance of airflow, which can be either natural or forced by fans. Additionally, although minimal, some heat is radiated from the heat sink into the environment. With the addition of the heat pipe with heat sink the efficiency of heat transfer to the surroundings becomes more efficient.^77^ The fast charging of lithium battery packs become highly effective with use of an optimized heat pipe thermal management system.^78^ According to Tran *et al.*,^79^ adding a heat pipe to a battery module lowers the heat sink's thermal resistance by 20% when cooling with low air velocity and 30% when cooling naturally. Egab and Oudah^80^ used a variety of heat sink configurations, such as regular heat sinks, perforated heat sinks, and heat sinks with a sequence of dimples in different shapes, such as circular, elliptical, and cylindrical, to explore the thermal management of cooling systems. Circular holes are drilled along the fins of the perforated heat sink, allowing air to enter *via* the holes. According to the findings, a traditional heat sink without dimples raised the battery temperature by 1.5 °C. In contrast, a heat sink with cylindrical dimples did not. Moreover, adding a dimpled-perforated heat sink resulted in a 3 °C drop in temperature under the same conditions. The system design used by the Egab and Oudah is shown in Fig. 3.

Mohammadian *et al.*^81^ found that the use of pin-fin heat sinks in combination with porous metal foam significantly enhances thermal performance by reducing battery temperature. The most effective configuration involved aluminium pin fins integrated with porous aluminium foam, which not only lowered the temperature but also improved temperature uniformity, making it a promising solution for the thermal management of lithium-ion batteries. In a related study, Hosseinirad *et al.*^82^ investigated the performance of fluid-based heat sinks with various fin geometries. Their analysis focused on four primary designs: straight–straight–straight (SSS), wavy–wavy–wavy (WWW), straight–wavy–straight (SWS), and wavy–straight–wavy (WSW). To optimize both pressure drop and base temperature, they implemented four different interruption arrangements across the heat sink configurations. The results show that wavy structures, particularly the WWW design, provide the best battery thermal performance but occupy more space. However, the introduction of interruptions is more effective in straight-fin models (SWS and WSW), enhancing both thermal and hydrodynamic performance. The interruption improves performance across all models except for WWW. Refrigerants used in heat sinks for battery thermal management systems play an important role in transferring heat away from battery cells, preventing overheating, and ensuring optimal performance. The choice of refrigerant is influenced by factors such as thermal conductivity, boiling point, environmental impact, and safety. Kang *et al.*^83^ emphasized the importance of refrigerant properties in battery thermal management systems, considering both cooling performance and environmental impact. They explored both single and mixed refrigerants, noting that R1234yf and R152a could serve as viable replacements for R134a, as R134a has a high global warming potential. While R744 offers a strong heating capacity, its limitations in certain applications should be carefully considered. Mixed refrigerants can effectively reduce global warming potential and flammability, leading to improved system efficiency. The flow of hydrofluoroolefin refrigerant (R1233zd(E)) in a parallel mini-channel heat sink was investigated by Dan Xu *et al.*.^84^ They discovered that higher heat flux and lesser subcooling can cause flow instability, impacting the effectiveness of battery thermal management systems. To increase stability, they suggest modifying the heat flux and inlet subcooling conditions.

### 3.2 Active thermal management techniques

**Fig. 4:**
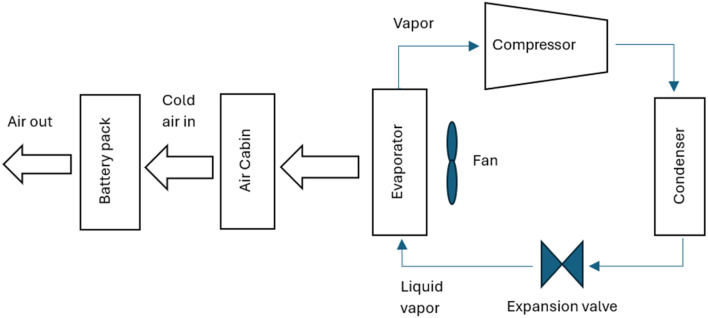
Air cooling system of the battery by vapor compression refrigeration cycle.

**Fig. 5:**
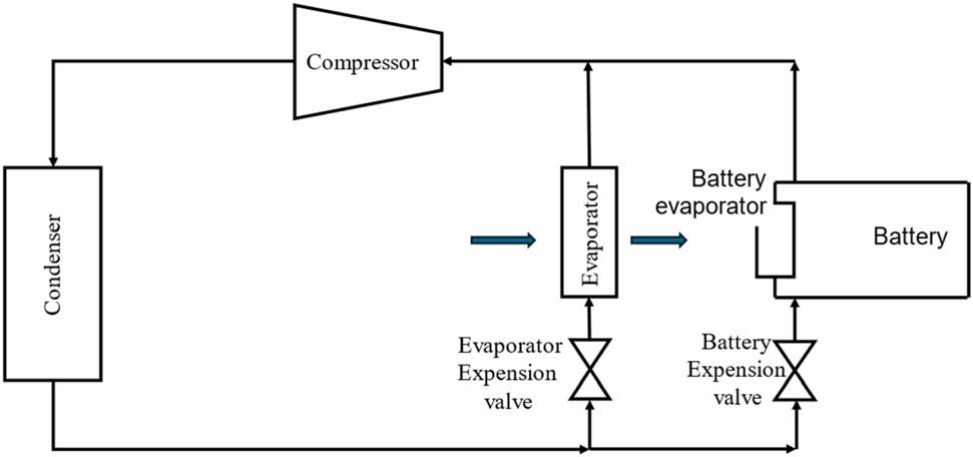
Active cooling system by refrigerant.

**Fig. 6:**
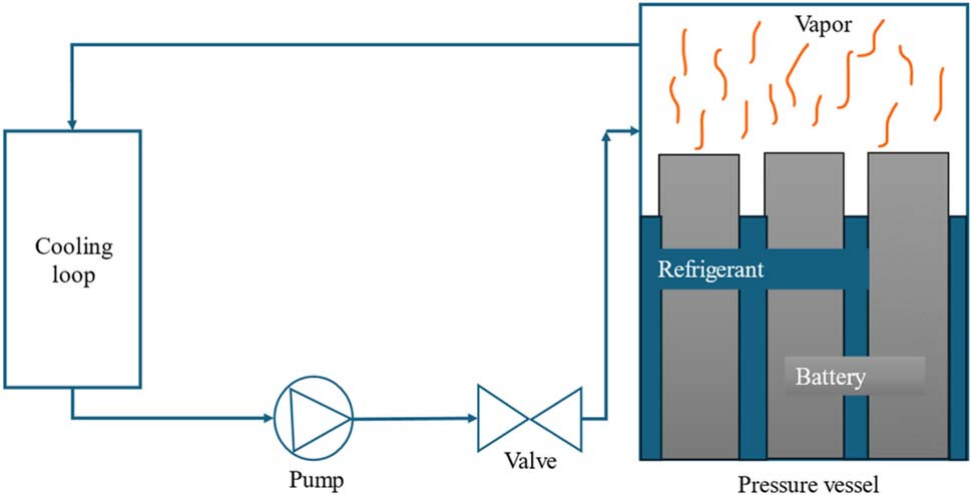
Immersion cooling method.

**Fig. 7:**
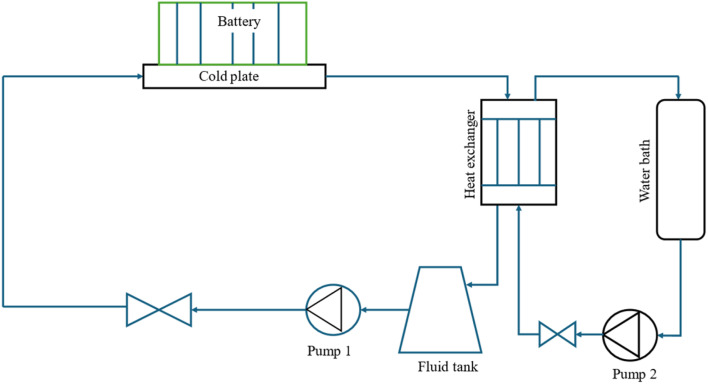
Battery cooling system by cold plate.

**Fig. 8:**
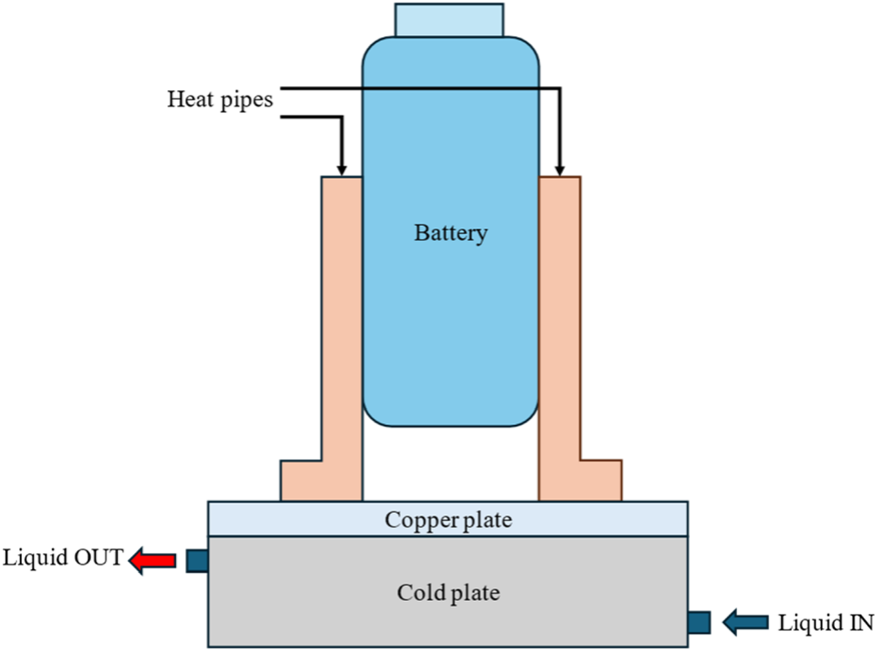
Battery cooling system using heat pipe coupled with cooling plate.

Active thermal management strategies for battery systems include several approaches designed to maintain optimal operating temperatures. Air cooling is a common method, where fans are used to dissipate heat from the battery modules, although its effectiveness may be limited in high-temperature environments. Liquid cooling, another widely used strategy, involves circulating fluids such as water, glycol, or dielectric fluids around the battery pack for efficient heat absorption and transfer. Refrigerant cooling uses a refrigeration cycle to lower battery temperatures, providing precise control and increased cooling capacity, particularly for high energy density applications. It is a versatile and compact method of cooling the battery, offering greater flexibility compared to fan and duct systems. By connecting the battery's evaporator in parallel with the cabin's evaporator within the same refrigeration loop as shown in Fig. (2), both components can operate efficiently under a single cooling system.^85^ This setup could enable effective thermal management for both the battery and the vehicle cabin without the need for separate cooling systems. Heat pipe systems combine passive and active cooling, using heat pipes to transfer heat away from specific areas of the battery pack while an external cooling system helps to manage the overall temperature. These strategies, either individually or in combination, play a crucial role in ensuring the safety, performance, and longevity of batteries in electric vehicles. The active method is employed when heat cannot be sufficiently removed by natural convection. An active system can be integrated into a variety of devices and applications, withstand large heat loads, and be precisely managed to maintain temperature ranges. However, the drawbacks associated with this technology include weight and power requirements and complex systems. These generally involve a high initial and maintenance cost due to the impacts on the overall operating system, which is not always optimal for manufacturers.^86^ In applications with high current demands, active thermal management systems also often face challenges in keeping cell temperatures within safe limits. They also tend to have issues with temperature unevenness across both cell and module levels.^87^ To address these limitations, some researchers integrate active and passive cooling methods, aiming to capture the benefits of each while minimizing the complexities of using an active system. Zhao *et al.*^88^ used a composite PCM made of from copper and foam in a effort to integrate active and passive cooling systems. In this hybrid cooling system, a coolant is circulated through tubes. The addition of copper foam significantly improved thermal management, reducing the battery surface temperature by 14 °C compared to a system using pure PCM alone. Choi and Yao^89^ investigated a battery cooling system using electrolyte circulation along the plates of a lead–acid battery, finding it to be the most effective active cooling method for removal of heat to ensure a uniform temperature distribution. The effectiveness of vortex generators in enhancing heat transfer within active thermal management system is investigated by Mondal *et al.*^90^ Small, fin-like objects called vortex generators are positioned in the flow channels. Their goal is to produce swirling or vorticous flows that improve the mixing of fluid close to the channel walls, resulting in improved heat transmission. To determine which vortex generator winglet configuration is optimal for thermal management, the study tests four distinct winglet configurations. The study concludes that vortex generators are an effective means of enhancing temperature uniformity and heat transfer rates in lithium-ion battery modules, thereby improving the overall efficiency of the cooling system. The study finds the ideas for enhancing BTMS with liquid-based system by examining different setups. Some modern EVs and HEVs, such as the Tesla Model S and Model 3, BMW i3 and i8, and Chevrolet Volt, utilize an active liquid-based cooling systems.^91^ These systems offer to vehicles the high heat transfer efficiency. Wu *et al.*^92^ reviewed the active liquid-based systems, focusing on both direct and indirect contact modes. They concluded that the heat pipe based BTM are most efficient since they utilize phase changes from liquid to gas improving overall heat transfer efficiency. The energy efficiency of the system can be further improved by integrating it with subsystems like HVAC which utilize liquid recirculation across the vehicle's thermal management systems. Saw *et al.*^13^ proposed mist cooling as an effective thermal control strategy for battery packs. They conducted both experimental and numerical investigations to compare the thermal performance of conventional dry air cooling with mist cooling. Simulation results indicated that the surface temperature of the battery module could be maintained below 40 °C using a mist cooling system with a mass flow rate of 5 g s^−1^ and a mist loading fraction of 3%. In a separate study, Basu *et al.*^91^ developed a three-dimensional electrochemical-thermal model to simulate the behaviour of a battery pack equipped with a liquid-cooled thermal management system under varying conditions, including different discharge currents and coolant flow rates. The model accurately predicted variations in the operating temperature of individual battery cells and revealed that contact resistance significantly impacts the pack's overall thermal performance. Hamut *et al.*^85^ conducted a comparative analysis of three thermal management strategies: passive cabin cooling using air, active liquid circulation with refrigerant, and active liquid circulation combining refrigerant and coolant. Among these, the hybrid active system (utilizing both refrigerant and coolant) demonstrated superior performance, achieving the lowest temperature rise (3.9 °C over 30 minutes), the highest cell temperature uniformity (median temperature difference of 2.5 °C), and the lowest entropy generation rate (0.0121 W K^−1^). Following figures show the line diagrams of different active cooling systems of battery pack. Fig. 4 illustrates a cooling system where air flows through the car cabin before being directed to the battery system and is then discharged into the atmosphere. In this setup, the cooling air is not recycled; instead, relatively low-temperature air is blown through ducts and manifolds by a fan to maintain the battery's thermal balance. In a system where air is recycled, heat is transferred from the relatively high-temperature air leaving the battery to the evaporator, allowing the cooled air to return and effectively absorb heat again.^93^Fig. 5 depicts a cooling configuration where the battery evaporator is connected in parallel to the evaporator in the vehicle's refrigeration loop,^85^ optimizing thermal regulation by integrating the battery cooling into the broader HVAC system for better energy distribution. Fig. 6 presents a schematic of immersion cooling, where the battery is submerged in a dielectric refrigerant for direct heat transfer,^83^ a method that ensures uniform cooling and reduces temperature gradients within the battery cells, thus enhancing safety and longevity. Fig. 7 showcases an indirect cooling approach using a cold plate, which facilitates heat removal *via* conduction through a thermally conductive interface, improving overall cooling efficiency while minimizing electrical interference.^94^Fig. 8 represents a thermal management system that incorporates heat pipes coupled with a cooling plate, using the high thermal conductivity of heat pipes to rapidly transfer heat away from critical battery areas, ensuring more stable temperature control and preventing hotspots.^95^

## 4. Integration of hybrid energy storage systems (HESS)

Hybrid Energy Storage Systems (HESS) have emerged as a versatile and efficient solution to address the growing demand for energy storage across multiple sectors, including electric vehicles, renewable energy integration, and grid stabilization. HESS involves the strategic combination of two or more energy storage technologies with complementary operational characteristics such as power density, self-discharge rate, efficiency, and lifespan to enhance overall system performance. By integrating various technologies, such as batteries, hydrogen storage, ultracapacitors (UCs), and thermo-mechanical energy storage, HESS can meet a wide range of energy demands, including electricity, heating, and cooling, within a unified system.^96^ In particular, the combination of batteries and supercapacitors facilitates the efficient integration of renewable energy sources into the power grid by balancing long-term energy storage requirements with short-term power quality and frequency regulation. Due to their ability to manage peak power demands while offering cost-effective, practical, and durable performance, hybrid systems are often preferred over single-technology energy storage solutions.^97^ A notable example includes the 1.2 MW hybrid UC-battery HESS installed by Duke Energy in North Carolina in 2016, which aids in peak demand response, load shifting, and solar power smoothing.^98^ A study conducted by Saw *et al.*^99^ evaluated the overall performance of Li-ion batteries integrated with a HESS under variable conditions. The results demonstrated improvement in overall performance of the battery due to reduced dynamic stress and optimized thermal regulation due to the incorporation of supercapacitors into the battery pack. Thermo-mechanical energy storage systems, as outlined by Wolf-Dieter Steinmann,^100^ work by converting mechanical energy into thermal energy and *vice versa*. There are three main types of thermo-mechanical energy storage systems discussed in this work the Compressed Air Energy Storage (CAES), Power to Heat to Power (PHTP), and Pumped Thermal Energy Storage (PTES). In CAES, pressurized air is stored and later expanded with the addition of heat during discharge. PHP systems store electrical energy as heat, which is then used to drive a thermal cycle during discharge. A variation of these systems, Power to Heat to Combined Heat and Power (PHCHP), not only powers a thermal cycle but also provides heat for other industrial applications. PTES uses excess electricity to create a temperature difference between heat reservoirs, enabling energy recovery through thermal cycles. PTES systems can store both heat and cold energy, making them highly versatile in different energy applications. Khaligh and Li^101^ studied HESS that combines batteries, UCs, and fuel cells, concluding that this configuration is better suited for advanced hybrid electric vehicles compared to single energy storage devices. Individual storage devices, such as batteries, UCs, and fuel cells, were found to be insufficient in meeting the full demands of advanced hybrid electric drivetrains. Thilo Bocklisch^102^ explores common applications of HESS focusing on how energy storage units are connected and managed by explaining basic energy management concepts, including hierarchical control optimization methods. Four HESS setups are discussed as suitable options for decentralized photovoltaic systems: (a) power-to-heat with a battery, (b) power-to-heat with battery and hydrogen storage, (c) supercapacitor with a battery, and (d) dual-battery setup.

### 4.1 Role of HESS in thermal management

HESS enhances thermal management by optimizing temperature regulation. In a system combining batteries, UCs, and fuel cells, the load on each component, especially the batteries, is reduced, which helps lower internal resistance and decreases heat generation within the batteries. Thermal performance of batteries is improved by energy storage with Li-ion batteries and supercapacitors.^99^ Giorgio *et al.*^103^ introduced an innovative on-board energy storage system concept that integrates a battery pack with a metal hydride tank. The core idea of this design is to use the exothermic absorption and endothermic desorption of hydrogen in metal hydrides for thermal management, utilizing these reactions to heat and cool the battery pack as needed to maintain optimal temperature control. For thermal management systems, some researchers employ the HESS. The comparison study between HESS and traditional battery systems for heat control is displayed in Table 8.

**Table 8:** Comparison of conventional battery system with HESS for thermal management

System type	Thermal management technique	Maximum temperature (°C)	Temperature uniformity (Δ*T*, °C)	Cooling efficiency/other benefits	References
Conventional (Li-ion)	PCM (capric acid, 3 mm thick)	32 °C (305 K)	Δ*T* ∼ 3–5 °C, typical for PCM	Effective at thin layers, but limited scalability	Verma *et al.* (2019)^65^
Conventional (Li-ion)	PCM (paraffin with graphene additives)	∼52 (inferred from enhanced PCM performance)	∼4–5 (typical for enhanced PCM)	Improved thermal conductivity with graphene, but scalability is limited	Cai *et al.* (2023)^104^
Conventional (Li-ion)	Air-based BTM with CNN-ABC and MPC control	∼40–45 (inferred from optimized air-based systems)	∼3–5 (inferred from advanced control uniformity)	Enhanced energy efficiency, real-time adaptability using deep learning and MPC	Ali *et al.* (2024)^105^
Conventional (Li-ion)	Forced air cooling	32.7	≤3	Enhanced cooling by optimizing air flow and layout	Gharehghani *et al.* (2024)^106^
Conventional (Li-ion)	PCM + CuO nanoparticles + extended surfaces (Fins)	↓16 (*vs.* PCM alone)	Not specified	Battery temp maintained below 60 °C for 41–93% longer; operational duration improved by 92.5%; fin length increase (6–14 mm) gives +13.62% time; improved heat spreading & thermal conduction	Shehabaz *et al.* (2025)^107^
Conventional (Li-ion)	Liquid cooling with optimized cold plate, jacket & channel design	27.8–40.0	0.8–11.0	Multiple configurations achieved <40 °C with Δ*T* as low as 0.8 °C; techniques include minichannels, jackets, baffles, U-turn and serpentine designs	Zhao *et al.* (2023)^108^
Conventional (air cooling BTMS III)	Forced air-cooling, dual outlets	51.4	1.78	6.72 °C *T*_max_ reduction, 8.91 °C Δ*T* reduction, lowest pressure drop (17.36 Pa), best thermal uniformity	Yang *et al.* (2023)^109^
Conventional (liquid cooling)	Liquid cooling external coolant channel	Not specified	0.8	Moderate cooling capacity, balanced design	Zhao *et al.* (2023)^108^
Conventional (liquid cooling (S-1, S-2, S-3))	Liquid cooling, serpentine channel with fin structure, with long fin structure and with cavity and rib structure	26.5, 27 and 25.7 respectively	2.0, 2.5, and 2.2	Higher pressure drop (200 Pa at 12 L h^−1^), lower heat transfer performance	Chen *et al.* (2023)^110^
HESS (H-HESS)	Microchannel heatsink and thermoelectric cooling	35–40 °C for H-HESS, 40–45 °C for BESS	3–5 °C for H-HESS, 5–8 °C for BESS	The H-HESS achieves a 55.7% peak current reduction and ≈2% improvement in battery capacity loss over 30 days	EIGhanam *et al.* (2023)^111^
HESS (battery + MH)	MH hydrogen storage with PCM (sandwiched MH-PCM unit)	∼45 (inferred from enhanced heat transfer)	∼1–2 (inferred from faster heat transfer)	77.8% faster hydrogen absorption, 58.8% faster desorption, improved heat transfer	Ye *et al.* (2021)^112^
Hybrid (hydrogen-powered)	Metal hydride reactor system (Hydralloy C2) using onboard H_2_ pressure	30 (ambient), 20 (cooling)	∼10 (inlet-outlet Δ*T* of HTF)	Cooling efficiency up to 81% with optimized valve switching; avg. cooling power of 662 W at 5 kW FC load; specific cooling power: 227 W per kg MH	Weckerle *et al.* (2020)^113^
HESS (battery + MH)	LiFePO_4_ battery with MH tank (electric scooter)	∼40–50 (inferred from passive cooling)	∼2–3 (inferred from passive cooling design)	Enhanced onboard energy density, passive thermal management	Di Giorgio *et al.* (2022)^47^
HESS (battery + MH)	Metal hydride (Hydralloy C5) integrated with Li-ion battery	Maintained near ambient (∼305 K); without TM > 330 K	Not explicitly stated; significant flattening of gradient	Endothermic H_2_ desorption enables passive thermal management, stabilizes battery temp, improves volumetric energy density, and extends runtime by ∼1 h; 16% of total H_2_ demand covered by HESS	Di Giorgio *et al.* (2023)^24^

**Fig. 9:**
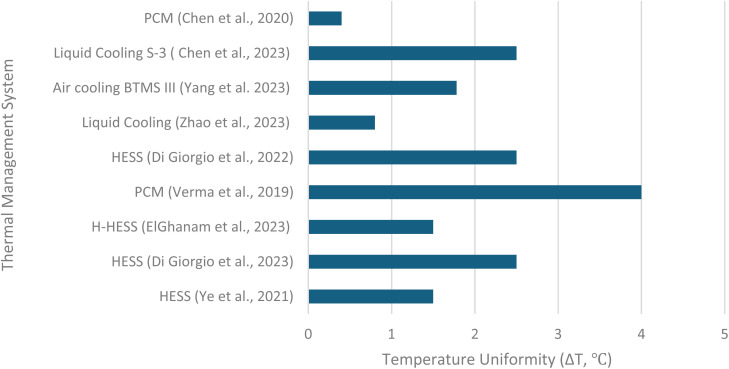
Temperature uniformity comparison (HESS *vs.* conventional systems).

HESS that uses MH for thermal management perform better than traditional lithium-ion battery systems, as shown by various studies. For example, Di Giorgio *et al.*^24^ studied a HESS using Hydralloy C5, which kept battery temperatures close to ambient levels (∼32 °C) compared to over 57 °C without cooling, with a very even temperature distribution (less than 3 °C difference). This is better than conventional PCM systems, which reached ∼52 °C with a 4–5 °C temperature difference (Cai *et al.*,^104^), and is comparable to forced air cooling, which hit 32.7 °C with a similar temperature difference (Gharehghani *et al.*,^106^). Similarly, Ye *et al.*^114^ combined MH with PCM in a HESS, achieving ∼45 °C with good temperature uniformity (1–2 °C difference), 77.8% faster hydrogen absorption, and 58.8% faster release compared to standard PCM systems (3–5 °C difference, Verma *et al.*,^65^) or air-cooling systems with advanced controls (3–5 °C difference, Ali *et al.*,^105^). Weckerle *et al.*^113^ showed a metal hydride system cooling to 20 °C (with ambient at 30 °C), achieving 81% cooling efficiency and 662 W cooling power, rivaling energy-heavy liquid cooling systems (27.8–40 °C, 0.8–11 °C difference, Zhao *et al.*,^108^). Unlike traditional systems that need complex additives (like CuO nanoparticles and fins, extending runtime by 92.5%, Shehabaz *et al.*,^107^) or active cooling, HESS uses the natural heat-absorbing process of hydrogen release for passive cooling, saving energy and simplifying design. For example, Di Giorgio *et al.*^24^ extended runtime by about an hour and met 16% of hydrogen demand, boosting energy density for applications like electric scooters benefits not seen in conventional systems. These studies show HESS provides cooler temperatures, more consistent heat distribution, and practical advantages, making it a strong option for efficient, scalable thermal management in electric vehicles and other uses. Fig. 9 compares temperature uniformity between HESS and conventional systems.

**Fig. 10:**
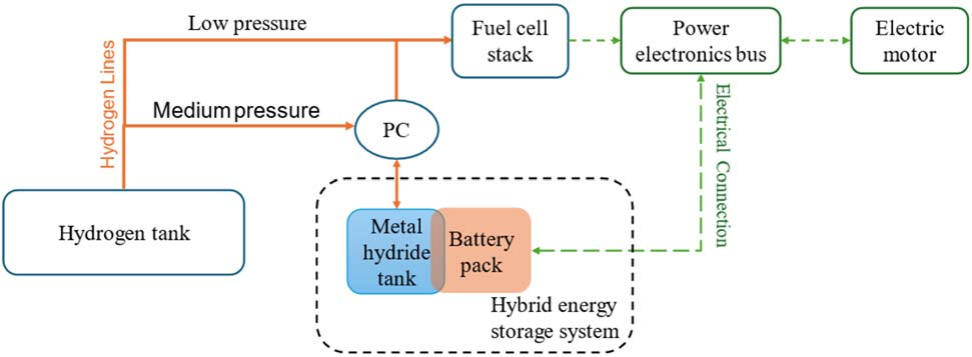
Working diagram of HESS with battery pack and metal hydride tank.

Fig. 10 presents the working diagram of the HESS, illustrating the integration of the battery pack with the metal hydride tank. In this system, when the battery pack temperature rises, the pressure controller (PC) permits hydrogen to flow from the MH storage system to the fuel cell stack. This creates a pressure drop in the MH storage, triggering hydrogen desorption and a subsequent temperature decrease, which cools the battery pack. Conversely, when the battery pack requires heating or the MH storage system needs recharging, the PC directs hydrogen from a high-pressure tank into the MH storage system. This causes a pressure increase within the MHs, inducing hydrogen absorption, which generates heat and warms the battery pack. According to Afzal *et al.*,^115^ the following equations control the heat transport related to hydrogen absorption and desorption inside the metal hydride bed:
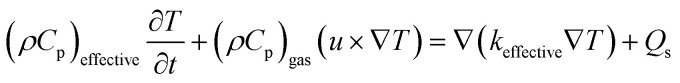
where,

4 (*ρC*_p_)_effective_ = *ε* × *ρ* × *C*_p_gas__+(1 − *ε*) × *ρ*_metal_ × *C*_p_metal__

5 *k*_effective_ = *ε* × *k*_gas_ + (1 − *ε*) × *k*_metal_

*C*_p_ = specific heat capacity, J kg^−1^ K^−1^, 
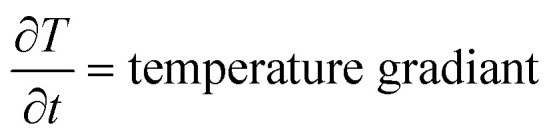
, *u* = velocity, m s^−1^, *k* = thermal conductivity, W m^−1^ K^−1^, *ε* = porosity, *Q* = heat source or sink, W m^−3^

**Fig. 11:**
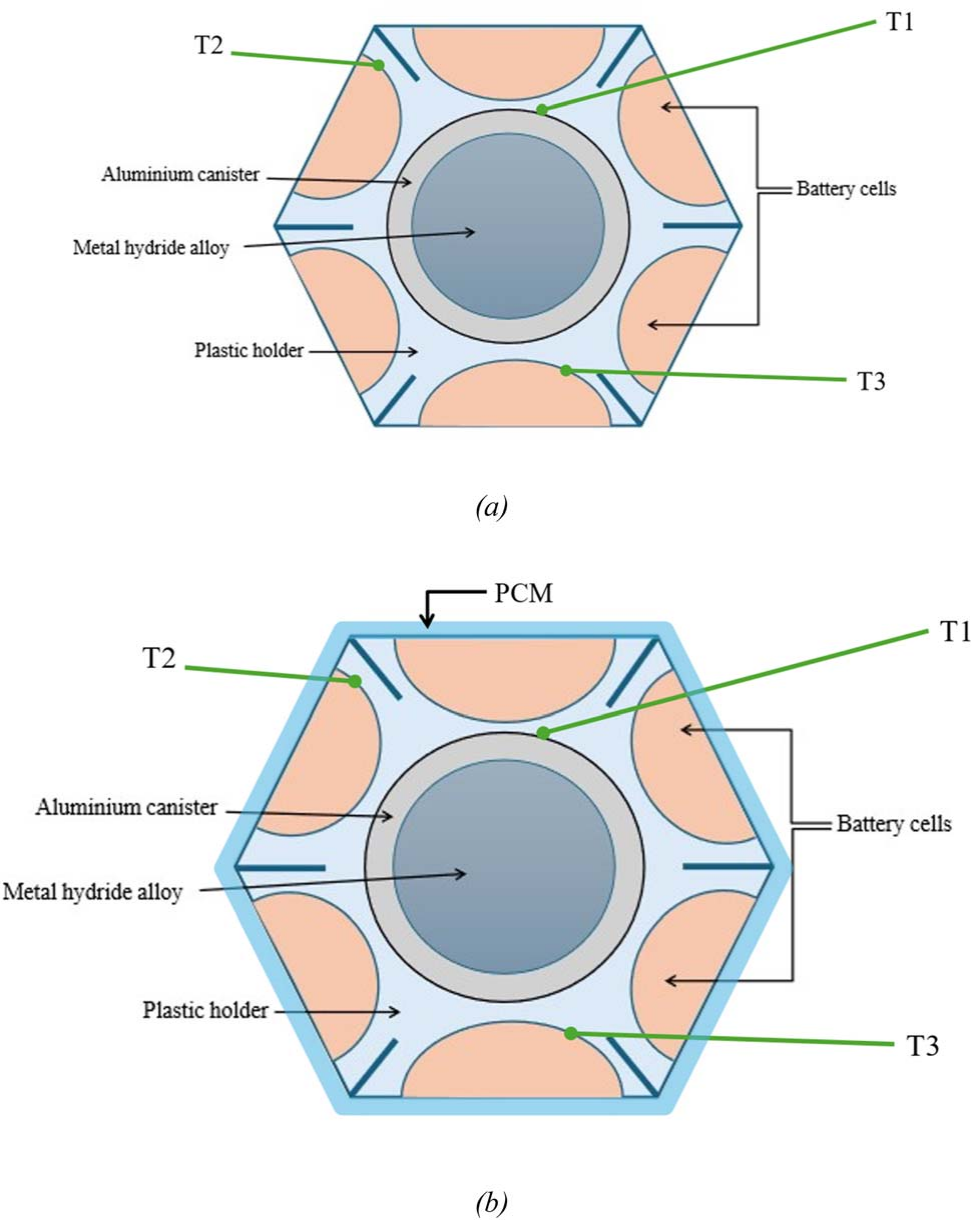
HESS (a) without PCM, and (b) with PCM.

Giorgio *et al.*^24^ have implemented a similar configuration for a plug-in electric scooter by replacing the battery pack with a HESS system. The improved systems gave promising results with the maximum temperature rise being 12 °C compared to 30 °C in the conventional system. The improved thermal management was due to hydrogen desorption in the metal hydride tank. In this configuration, six cylindrical batteries surround the aluminium cylinders which contain the MH alloys packed in the form of 0–2 mm metal flakes. The heat transfer between the battery cells and aluminium cylinders is ensured by using a 3D printed plastic holder made of thermally conductive polylactic acid. Additional measures are taken to minimize the risk of thermal runaway by adding insulation cavities to the plastic holder. A schematic of this HESS geometry is shown in Fig. 10. This system supports passive thermal management of the battery pack and enhances overall onboard energy density. A prototype of HESS that combines a battery pack with a MH hydrogen storage system for an electric scooter was developed by Giorgio *et al.*^47^ This electric scooter features a 48 V, 2 kW brushless DC motor and originally used a 48 V, 40 Ah lead–acid battery pack. In the vehicle's electric configuration, the lead–acid battery was replaced with a more compact, higher-performing 48 V, 20 Ah lithium iron phosphate (LiFePO_4_) battery, a proton-exchange membrane fuel cell stack, and an MH tank for hydrogen storage. Fig. 11(a) illustrates the layout of this two-wheeler HESS integration. The geometry shown is a hexagonal arrangement, with key components labeled: an aluminum canister containing the MH alloy at the center, surrounded by cylindrical battery cells, all held together by a plastic holder. The points *T*_1_, *T*_2_, and *T*_3_ are temperature measurement points within this geometry. *T*_1_ is likely positioned near the outer edge of the battery cells, closer to the external boundary of the hexagonal module, as indicated by the arrow pointing outward. *T*_2_ and *T*_3_ are positioned closer to the central MH canister at different radial distances within the module, as indicated by their arrows, which point toward the inner regions of the geometry. According to the Di Giorgio *et al.*,^103^ the temperatures at *T*_2_ and *T*_3_ (closer to the MH canister) increase more significantly (from 27 °C to 31 °C) compared to *T*_1_ (from 26 °C to 26.5 °C). This suggests that the battery cells near the centre are heating up faster due to the 1C discharge rate, likely because of higher heat generation and less effective heat dissipation near the core of the module. After 3000 seconds, *T*_2_ and *T*_3_ show a sharp decrease (from 31 °C to 24 °C), while *T*_1_ remains relatively stable (around 26.5–27 °C). This indicates that the MH system's cooling mechanism (endothermic desorption of hydrogen) is effectively reducing the temperature near the center of the module, where *T*_2_ and *T*_3_ are located. A 30–40% hydrogen desorption can lower the battery pack temperature by about 15 °C, which aligns with the observed drop at *T*_2_ and *T*_3_.

### 4.2 Coupling HESS with the PCM

**Fig. 12:**
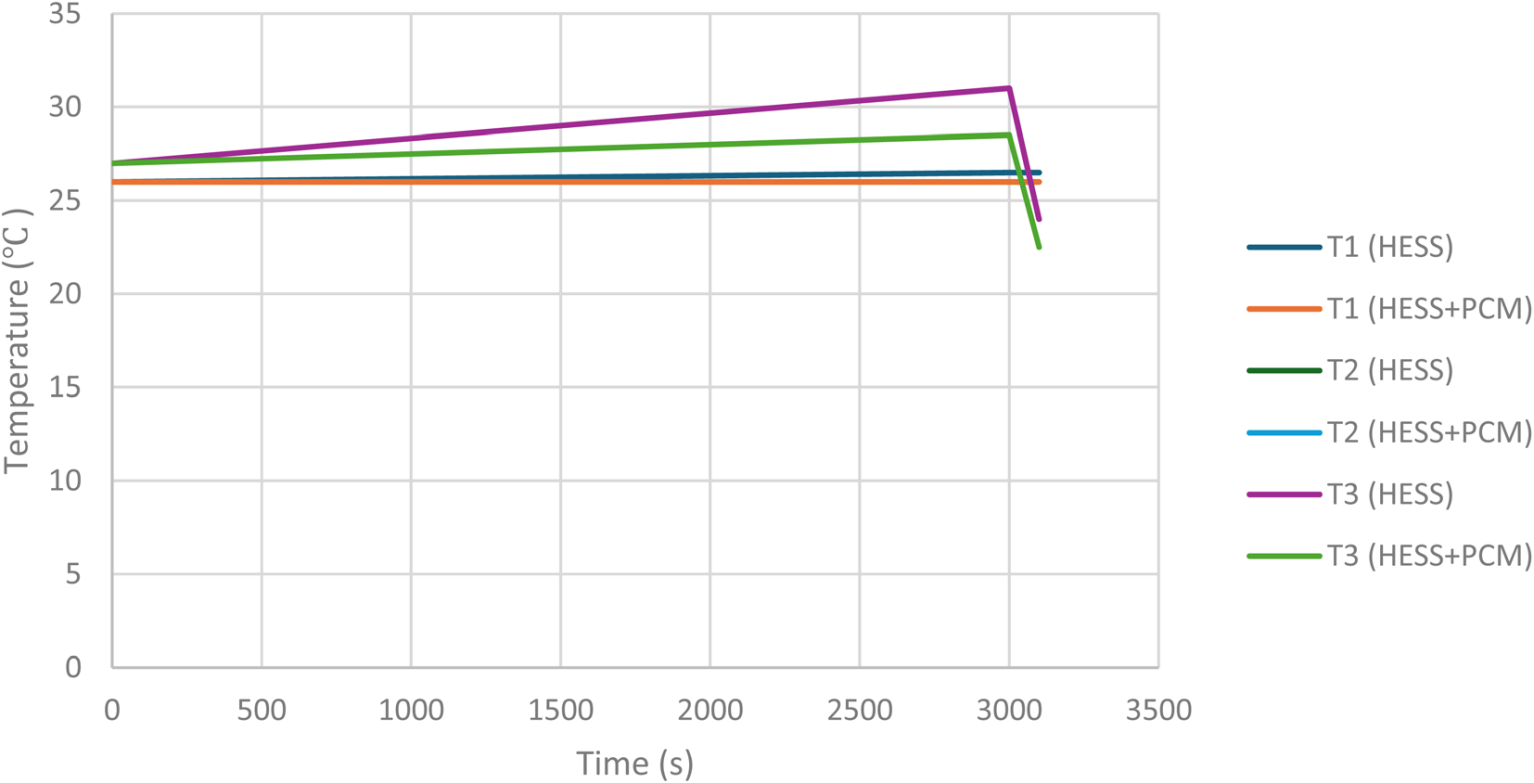
Comparison curve between all points *T*_1_, *T*_2_, and *T*_3_ of HESS with and without PCM.

Integrating phase change materials with a HESS that employs metal hydrides for hydrogen storage enhances thermal management by combining passive and active cooling mechanisms. PCMs, such as organic compounds like *N*-octadecane with a phase transition temperature of 25–30 °C,^67^ can be incorporated into the plastic holder or as a layer around battery cells to absorb excess heat during phase transition (*e.g.*, melting). As shown in Fig. 11, which shows the HESS with and without PCM, this integration significantly improves temperature control. Fig. 12 shows the comparison curves. During the initial temperature rise (27 °C to 31 °C at locations *T*_2_ and *T*_3_ according to Di Giorgio's^103^ study), the PCM absorbs latent heat, potentially capping the peak temperature at its melting point, around 28 °C. This could reduce the maximum temperature at *T*_2_ and *T*_3_ to approximately 28–29 °C instead of 31 °C. After 3000 seconds, when the MH system triggers hydrogen desorption, the combined effect of PCM and MH cooling could further lower temperatures at *T*_2_ and *T*_3_ to around 22–23 °C, compared to 24 °C with the HESS alone, due to enhanced heat transfer uniformity. At location *T*_1_, the PCM stabilizes the temperature more effectively, maintaining it at approximately 26 °C instead of 26.5 °C, by improving heat dissipation across the battery module. Thus, PCM reduces peak temperatures and enhances temperature uniformity across the battery pack, complementing the MH system's active cooling and preventing overheating of central battery cells (*T*_2_ and *T*_3_), which is critical for battery longevity and safety.

The addition of PCMs also reduces reliance on active hydrogen desorption, improving overall system efficiency.^116^ By absorbing heat during peak generation, PCMs can reduce the heat load on the MH system by 20–30%, as estimated from studies like Ling *et al.*^86^ For example, if the total heat load is 60 W, the PCM could handle 15 W, leaving the MH system to manage 45 W. With the MH system's cooling power at 40 W, this results in an efficiency of approximately 40/45 ≈ 89%. Additionally, PCMs minimize thermal gradients, reducing uneven battery degradation and indirectly enhancing long-term operational efficiency. However, incorporating PCMs slightly reduces the system's gravimetric and volumetric energy density due to added weight and volume. Assuming a PCM layer increases the system's weight by 10%, the gravimetric density may decrease to around 165 Wh kg^−1^, and the volumetric density may drop to approximately 480 Wh L^−1^.

The MH system in the HESS relies on a reversible reaction where gaseous hydrogen forms a metal alloy or intermetallic compound, offering advantages such as compactness, safety, and high volumetric hydrogen density, surpassing that of a 700-bar pressurized tank. This makes MHs ideal for compact system designs.^117^ Weckerle *et al.*^118^ demonstrated that MHs not only store hydrogen efficiently but also recover energy typically lost during high-pressure hydrogen compression (approximately 15% of hydrogen's lower heating value). By integrating MH-based reactors into an air-conditioning system that alternates between hydrogen absorption and desorption, this setup reuses compression energy for cooling, significantly enhancing efficiency in fuel cell vehicles. Muthukumar and Groll^119^ provided a comprehensive review of MH-based heating and cooling systems, emphasizing their application in air conditioning. The performance of these systems depends on MH properties, such as high enthalpy of formation, low specific heat, high hydrogen absorption capacity, and high thermal conductivity.

**Fig. 13:**
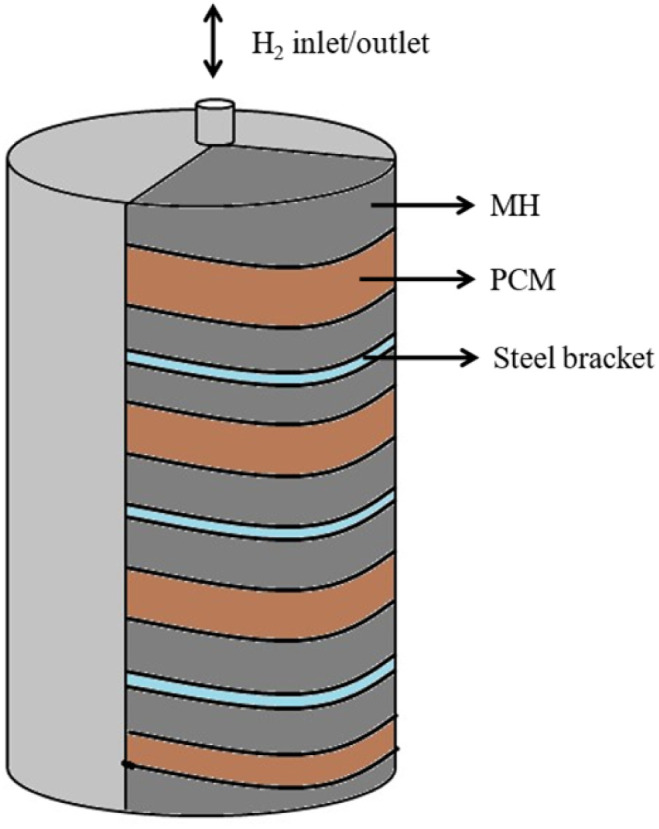
Storage tank with sandwiched MH-PCM unit.

Integrating PCMs with MH hydrogen storage tanks further enhances efficiency by recycling absorption heat as a heat source for desorption.^116^ Various techniques, including optimized reactor vessel shapes, heat exchangers, PCMs, cooling tubes, water jackets, nano-oxide additives, and high-thermal-conductivity additives, have been developed to improve heat transfer in MH systems.^120^ Ye *et al.*^114^ investigated PCM configurations in hydrogen storage tanks, finding that sandwiched MH-PCM units, as shown in Fig. 13, significantly improve heat transfer and reaction rates due to a larger heat transfer surface area and reduced thermal resistance. Their results showed a 77.8% reduction in hydrogen absorption time and a 58.8% reduction in desorption time. Zhu *et al.*^121^ introduced a thermal management approach combining an MH-based hydrogen storage tank with a proton-exchange membrane fuel cell, which reduces degradation in fuel cell hybrid vehicles during long-term operation. Effective heat transfer between components is critical for maintaining consistent hydrogen generation and supply rates from the MH tank to the fuel cell. Additionally, combining a battery with an ultracapacitor in the HESS enhances performance compared to a battery-only system, effectively managing battery temperature and improving energy storage capabilities. Hoelscher *et al.*^122^ explored this hybrid system, demonstrating its versatility for hybrid electric vehicles through both active and passive thermal management. By integrating PCMs with MH-based cooling and ultracapacitors, the HESS achieves superior thermal regulation, energy efficiency, and system durability, making it a robust solution for advanced energy storage applications.

Zhou *et al.*^123^ proposed an alternative hybrid approach for battery thermal management, incorporating a PCM/copper foam combination along with an air jet pipe and liquid cooling channel for cylindrical Li-ion batteries. Their findings showed that with this system, the Li-ion battery reached maximum temperatures of 19.1 °C and 35.6 °C at ambient temperatures of 15 °C and 35 °C, respectively. Ling *et al.*^124^ introduced a system that combines PCM with forced air convection. The PCM used in this setup, RT 44HC/EG, was positioned around the battery. The battery module, encased in PCM, was then placed in an air tunnel with a rectangular cross-section. This system effectively maintained the battery pack's maximum temperature below 50 °C in cycles with discharge rates up to 2C, even with a 7 °C rise in ambient temperature. Kiani *et al.*^125^ also performed experimental and numerical analysis to design a thermal management system combining an active cooling system that uses alumina nanofluid with a passive cooling method that uses paraffin-saturated copper foam as the PCM. The findings demonstrated that nanofluid cooling successfully maintains safe battery operation under high-stress circumstances and increases the battery's operational time when compared to water-based cooling. So, these are the advanced methods for enhancing energy storage and controlling battery temperature. Some experimental studies on sophisticated thermal management techniques carried out by researchers are shown in Table 9.

**Table 9:** Experiments on BTMS

References	Thermal management technique	Experimental conditions	Key findings	Advantages
Cen *et al.*^126^	BTMS using EV air conditioning refrigerant with finned-tube heat exchanger and aluminium frame	Extreme ambient temperature of 40 °C; discharge rates of 0.5C, 1C, 1.5C in laboratory tests, and road drive tests	Maintained the temperature of the battery pack within the predetermined range; decreased the temperature inconsistency in the battery pack	Effective cooling; improved temperature uniformity with less than 4 °C difference in lab tests and 1.5 °C in road tests
Lazrak *et al.*^127^	Paraffin RT35 (PCM) integrated in BTMS	PCM around the cell in a Plexiglas with finding the temperature at 4 different depths	Temperature increases near the cell by over 5 °C and achieves a more uniform temperature distribution around the cell	Enhanced temperature control and uniformity; improved energy performance
Oh *et al.*^128^	Used various PCMs including RT15, RT31, EG5, EG26, and composite PCM (paraffin with graphite)	Used lithium polymer pouch battery with and without PCM; simulation with CFD software (FLUENT)	EG26 PCM reduced battery surface temperature to 30.1 °C from 37.6 °C without PCM; composite PCM exhibited enhanced cooling and temperature uniformity; CFD resulted matched experimental data with 1–2 °C deviation	Improved temperature control and uniformity; enhanced heat transfer rate; maintained safe operating conditions and reduced voltage drop with expanded graphite PCM during discharge
Xu *et al.*^129^	Hybrid cooling system with composite silica gel plate (CSGP) and copper cooling tubes (CSGP-LC)	Battery module with CSGP and copper tubes; natural cooling, forced convection (CSGP-FC), and liquid cooling (CSGP-LC) modules; testing at 4C discharge rate, 0.8 m s^−1^ water flow rate; 10 charge/discharge cycles	CSGP-LC maintained maximum temperature below 42.7 °C with temperature difference within 2.7 °C; energy consumption of CSGP-LC was half of CSGP-FC; efficient heat transfer through EG and copper foam addition to CSGP	Effective temperature control and stability, especially at high discharge rates; reduced energy consumption; superior thermal conductivity due to EG and copper foam
Li *et al.*^130^	Immersion cooling BTMS using silicone sealant (SS) and boron nitride (BN) composite (SS/BN)	18 650-type Li-ion battery module; comparison of air-cooled, pure SS, and SS/BN modules; tested under natural air cooling, forced ventilation, and immersion cooling at 3C discharge rate	SS/BN module reduced maximum temperature below 35 °C and temperature difference within 0.5 °C during 3C discharge; provided effective heat dissipation and corrosion protection	Simple structure; effective cooling and temperature uniformity; long-term corrosion protection, especially under water or moisture conditions
Wang *et al.*^131^	Heat pipe-based BTMS for cooling and heating	Experimental setup with heat pipe cooling/heating for battery cells at 2.5 to 40 W per cell; tested under 1–4 C discharge rates, at high thermal load conditions, and sub-zero temperatures (−15 °C to −20 °C for over 14 hours)	Heat pipes maintained battery surface temperature under 40 °C for <10 W per cell; effectively reduced temperature to 70 °C under thermal abuse (20–40 W per cell); maintained temperatures of 30.5 °C (3C) and 41.1 °C (4C) *vs.* 41.2 °C and 55.3 °C without cooling; functioned well after repeated exposure to sub-zero climates	Effective cooling/heating under extreme and sub-zero conditions; durable heat pipe performance at −15 °C/−20 °C; rapid temperature recovery from frozen state; highly reliable for aggressive driving conditions and varied thermal scenarios
Rao *et al.*^132^	Oscillating heat pipe (OHP)-based battery thermal management (BTM) system	Battery and OHP positioning experiments; tested for impact of battery placement near OHP condensation section; evaluated horizontal *vs.* vertical OHP placement for reflow resistance; assessment of start-up temperature requirements	Placing battery near OHP condensation section reduced maximum battery temperature; vertical positioning of OHP decreased reflow resistance	Extended cycle life of battery by reducing temperature extremes; effective high-temperature heat transfer through OHP; improved EV performance through enhanced battery temperature control
Mbulu *et al.*^133^	Heat pipe-based BTMS with high Input power	L- and I-shaped heat pipes with water coolant; input power levels of 30, 40, 50, and 60 W; condenser sections cooled at flow rates of 0.0167, 0.0333, and 0.05 kg s^−1^	Maintained *T*_max_ below 55 °C and Δ*T* below 5 °C even at 60 W; transferred over 92.18% of generated heat	Effective temperature control under high heat loads; high heat transfer efficiency; suitable for high-power applications in EV battery packs

## 5. Economic and safety considerations

The integration of PCMs with hybrid energy storage systems utilizing metal hydrides enhances thermal management but possible economic and safety challenges must be addressed for successful commercialization and practical implementation of these technologies. This section provides a systematic comparison of thermal management techniques, evaluates commercialization barriers such as cost and volumetric constraints, and discusses safety and operational challenges, including hydrogen safety protocols and MH degradation. To enhance clarity, Table 10 compares PCMs, liquid cooling, and air cooling based on cost, efficiency, and application scenarios. PCMs offer passive cooling through latent heat absorption, effectively maintaining temperature uniformity in compact HESS for hybrid electric vehicles (HEVs).^134^ Liquid cooling provides superior cooling capacity but requires complex infrastructure, increasing costs.^135^ Air cooling is cost-effective but less efficient for high-power applications due to limited heat transfer rates.^136^ PCMs, with moderate costs and high efficiency, complement MH cooling, making them suitable for HEVs.

**Table 10:** Comparison of thermal management techniques based on cost, efficiency, and limitations

Technique	Cost ($ per kW)	Efficiency	Application	Limitations
PCM	50–100	High (latent heat)	HEVs, compact systems	• Volume change after phase shift may cause leakage
				• Added weight
				• Low thermal conductivity and temperature sensitivity
Liquid cooling	150–300	Very high	High-power systems	• Complex design, high cost, and added weight
				• Strict sealing to prevent coolant intrusion
				• Requires pumps and active cooling systems
Air cooling	20–50	Moderate	Low-power systems	• Low thermal conductivity of air; weak temperature control and poor uniformity
				• High power use and space demand in active cooling; reduces energy density
				• Limited improvement potential; not ideal for high energy density or fast charging

Commercializing HESS with PCM integration faces challenges related to cost, volumetric efficiency, and scalability. Organic PCMs, such as *N*-octadecane, cost approximately $2–10 per kilogram, contributing 5–10% to the system's total cost.^137^ The addition of PCM layers increases system weight and volume, reducing gravimetric and volumetric energy densities to approximately 165 Wh kg^−1^ and 480 Wh L^−1^, respectively, for a 10–15% weight increase.^138^ This trade-off can limit applications in space-constrained HEVs. Modular designs, allowing flexible PCM integration, can balance thermal performance and energy density. Recycling PCMs and MHs through thermal or chemical processes can reduce lifecycle costs by 15–20%. For instance, recycling MHs like LaNi_5_ retains 90% hydrogen storage capacity after 1000 cycles, enhancing economic viability.^139^ Practical implementation of HESS with MH and PCM integration involves safety and operational challenges. Hydrogen safety is critical due to its flammability, but MH systems mitigate risks by storing hydrogen in a solid state, reducing leak hazards compared to 700-bar tanks.^140^ Robust containment and real-time monitoring systems are essential to prevent hydrogen release during thermal runaway. MH degradation, caused by lattice strain and contamination, reduces hydrogen storage capacity by 10–15% after 5000 cycles. The energy penalty for managing hydrogen storage, including heat for desorption, consumes 5–10% of the system's energy output. PCM integration reduces this penalty by recycling absorption heat for desorption, improving efficiency by 20–30%. Advanced MH alloys, such as TiMn_2_-based compounds, offer higher thermal conductivity and cycle stability.^139^ Combining HESS with ultracapacitors further enhances energy management and reduces thermal stress, improving safety and efficiency.^141^ By addressing these economic and safety considerations through comparative analysis, cost-effective material strategies, and robust safety protocols, HESS with PCM and MH integration can achieve practical viability for advanced energy storage in HEVs and beyond.

## 6. Challenges and future directions

### 6.1 Current challenges and limitations

Current battery thermal management systems in EVs often face significant challenges when operating under extreme conditions, such as high ambient temperatures or high-power demands. These factors can lead to overheating, reduced battery performance, and potential safety risks.^142^ Key materials and technologies, such as PCMs and heat pipes, still exhibit limitations: PCMs often have low thermal conductivity and concerns about cost and scalability persist.^143^ Similarly, heat pipes face limitations during rapid heat fluctuations and in adverse environmental conditions, which may affect their reliability. Integrating Li-ion batteries with hydrogen storage systems or other hybrid energy solutions poses additional challenges. These hybrid systems require sophisticated control mechanisms, optimal energy balancing, and compatibility between storage media. Ensuring both stable and efficient operation of battery and hydrogen components within a single system, especially under dynamic EV driving conditions, remains complex. Additionally, these hybrid setups' costs and spatial requirements often limit their feasibility in commercial EVs. Advanced cooling methods, including immersion, fluid, and PCM-based, introduce further reliability and safety challenges. These methods address potential issues such as leakage,^144^ long-term instability, and corrosion.^145^ The compact design required for EVs makes it difficult to integrate large cooling components, such as fans, pumps, and additional fluid reservoirs, which can occupy valuable space within the vehicle.^146^ The power consumption of these thermal management systems can also directly affect the EV's range and efficiency, with high energy requirements potentially reducing driving range, especially for long-distance or high-power applications. Furthermore, innovative materials like metal hydrides, expanded graphite, and high-conductivity PCMs offer promising advancements for BTMS but come with high costs and manufacturing challenges, particularly at scale. The challenge remains to identify materials that balance thermal performance, durability, cost-effectiveness, and availability to meet the demands of commercial EV applications.

### 6.2 Future research directions

Research in BTMS holds great potential for enhancing efficiency, reliability, and safety in electric vehicles. To advance the development of BTMS technologies, the following research directions are promising:

Artificial intelligence (AI) enables machines to mimic human thinking and actions, and researchers are increasingly applying AI techniques, such as artificial neural networks (ANNs), to enhance battery thermal management systems in EVs. These systems are crucial for maintaining safe temperatures in lithium-ion batteries, ensuring both efficiency and longevity. For instance, Fang *et al.*^147^ developed an ANN model using a back-propagation network trained with the Levenberg–Marquardt algorithm to predict the surface temperature of nickel–metal hydride batteries, achieving high accuracy and suggesting its potential for LIBs. Similarly, Jiang *et al.*^148^ employed two types of recurrent neural networks (RNNs), long short-term memory (LSTM) and gated recurrent unit (GRU), to estimate LIB temperatures during discharging under varying ambient conditions, achieving real-time predictions with a maximum error of about 0.75 °C and a correlation coefficient above 0.95. Jaliliantabar *et al.*^149^ created an ANN model to predict LIB temperatures in a BTMS equipped with phase change materials (PCMs), demonstrating reliable performance across different operating conditions. Afzal *et al.*^150^ explored ANN models to predict the average Nusselt number, a key indicator of heat transfer, finding that deep neural networks with sigmoid and Gaussian activation functions outperformed single-layer models. Additionally, Chen *et al.*^151^ developed a convolutional neural network (CNN) to estimate battery state-of-health (SOH), achieving robust results with errors typically within ±2% for batteries above 80% SOH. AI and machine learning (ML) enhance BTMS by enabling real-time cooling adjustments, predicting temperature changes, preventing thermal runaway, and optimizing HESS under diverse conditions. Depending on the battery's operating conditions, machine learning algorithms can be trained to forecast the ideal PCM melting and solidification temperatures. Li *et al.*^152^ emphasized ML's role in improving LIB thermal safety and BTMS design, proposing future advancements like digital twin modeling for virtual system optimization. Looking ahead, self-learning algorithms could further adapt thermal management to varying driving scenarios, enhancing EV battery performance and safety.

Future advancements in BTMS need to focus on integrating advanced materials such as use of additives in PCMs, hybrid cooling technologies, and energy storage solutions to enhance thermal stability.^153^ Materials with self-healing capabilities or built-in corrosion resistance could further extend the durability and reliability of BTMS components. Additionally, hybrid cooling systems that combine passive strategies (*e.g.*, PCMs, heat pipes) with active methods (*e.g.*, forced air, liquid cooling) and potentially incorporate metal hydride hydrogen storage could achieve high-performance cooling with reduced energy consumption, addressing both thermal management and environmental sustainability.^154^ Furthermore, integrating Hybrid Energy Storage Systems with BTMS could enable self-regulating temperature control and optimized energy distribution based on real-time thermal demands, enhancing battery lifespan, stability, and overall system efficiency.

## 7. Conclusion

This review has comprehensively examined the status and advancements in battery BTMS and the integration of HESS for EV applications. The key findings and insights drawn from this study are as follows:

• Li-ion batteries remain the most preferred choice for EVs due to their high energy density, lightweight nature, and compatibility with both hybrid and fully electric vehicles. However, maintaining optimal temperature conditions is crucial for ensuring their long-term durability, safety, and performance.

• Several thermal management strategies have been explored in this paper, including the use of PCMs, heat sinks, and hybrid cooling systems. These approaches demonstrate significant potential in mitigating temperature fluctuations and enhancing battery lifespan, particularly under high-power operations and extreme environmental conditions. While PCMs offer passive thermal regulation and eliminate the need for continuous active cooling or heating, their low thermal conductivity poses a limitation. Nevertheless, their integration reduces system complexity and operational energy consumption.

• The integration of Li-ion batteries with hydrogen storage systems, particularly using metal hydrides, presents a promising path for improving thermal stability and energy efficiency. This hybrid approach not only enhances thermal control *via* endothermic and exothermic hydrogen absorption reactions but also supports optimized energy distribution within the battery pack. Despite these benefits, challenges related to cost, system complexity, and material compatibility continue to hinder widespread commercial adoption.

• Emerging technologies, including the application of advanced materials and AI, offer transformative potential for BTMS enhancement. AI-driven systems can enable real-time monitoring, predictive control, and dynamic thermal management, while innovations such as self-healing and corrosion-resistant materials can significantly extend system lifespan and reliability. Hybrid cooling approaches that combine passive and active methods are particularly effective in delivering balanced thermal performance.

• A notable highlight of this review is the effectiveness of composite PCMs, especially those enhanced with 12% expanded graphite, which achieved up to 30% reduction in temperature gradients compared to conventional PCMs ensuring better thermal uniformity and improved battery safety during high-rate operations such as fast charging.

• The proposed HESS framework integrating Li-ion battery packs, metal hydride tanks, and PCMs offers a passive thermal management solution that utilizes the latent heat characteristics of PCMs and thermochemical properties of hydrides. This system addresses the conductivity limitations of paraffin-based PCMs (∼0.2 W m^−1^ K^−1^) while reducing the need for energy-intensive active cooling mechanisms.

• Studies show that use of advanced materials significantly outperform conventional PCMs like paraffin wax, for example a 3 mm layer of capric acid PCM, can reduce battery temperature to 305 K. Additionally, integrating PCMs with heat pipes achieved 86.7% cooling efficiency while minimizing 11.7% heat loss, reinforcing the potential of hybrid thermal management systems.

• Further, the evaluation of sodium-ion batteries as an alternative to Li-ion technology reveals additional opportunities for HESS integration. With over 5000 cycles at 87.5% capacity retention, sodium-ion batteries offer improved safety and cost-effectiveness, aligning with the evolving demands of sustainable EV systems.

Looking ahead, future research should prioritize the development of high-performance eutectic PCMs with tailored melting points and superior thermal conductivity, as well as the construction of multiscale thermal simulation models to accurately capture heat distribution across battery packs under various operating conditions. Investigating the compatibility of HESS architectures with next-generation battery technologies (*e.g.*, solid-state batteries), along with long-term testing under real-world mechanical and thermal stress, will be essential for practical deployment. These advancements will not only enhance the safety, efficiency, and sustainability of EV battery systems but also support the global transition to net-zero transportation, in alignment with the United Nations Sustainable Development Goals (SDGs).

## Conflicts of interest

Authors declare no conflict of interest.

## Data Availability

No primary research results, software or code have been included, and no new data were generated or analysed as part of this review.
